# Authors' reply

**DOI:** 10.4103/0970-1591.33740

**Published:** 2007

**Authors:** Rakesh Kapoor, Pratipal Singh

**Affiliations:** Department of Urology, Sanjay Gandhi Post Graduate Institute of Medical Sciences, Lucknow, India. E-mail: rkapoor@sgpgi.ac.in

Dear Sir,

We greatly appreciate your interest in paper and your thoughtful queries.[1] It is true that GFR measured by radionuclide scans is not the best method (in comparison to direct assessment of creatinine clearance of renal moiety through percutaneous nephrostomy), however, in clinical practice it is the best noninvasive method to calculate GFR. Therefore most studies in the literature have taken it into consideration for treatment planning.

Improvement in function is one of the objective criteria we used to asses the success of the procedure as these are poorly functioning moieties and t½ on renal scan is difficult to comment in this situation. However, we agree with you that t ½ could have been used as a marker of success if possible.

Definition of poorly functioning kidneys (GFR < 25 ml/min) used is appropriate and in accordance with the existing literature and GFR < 10 ml/min or less than 15% relative function is currently recommended as the limit to consider nephrectomy in most series.[2]

Patient who underwent laparoscopic pyeloplasty in renal moiety with zero per cent function is asymptomatic till last follow-up, however, has not undergone renal scan in follow-up.

We feel that these patients with poorly functioning kidneys are best followed up with renal scan, as these moieties are not visualized on intravenous urography in our experience and intravenous urography is associated with the risk of radiation.
